# Hypermobile type Ehlers‐Danlos syndrome associated with hypogammaglobulinemia and fibromyalgia: A case‐based review on new classification, diagnosis, and multidisciplinary management

**DOI:** 10.1002/ccr3.2070

**Published:** 2019-02-19

**Authors:** Wei Zhang, Kevin Windsor, Richard Jones, David Oscar Taunton

**Affiliations:** ^1^ Internal Medicine Residency Program Brookwood Baptist Health Birmingham Alabama; ^2^ Alabama Oncology at Grandview Medical Center Birmingham Alabama; ^3^ Clinic for Rheumatic Diseases Tuscaloosa Alabama

**Keywords:** chronic pain, fibromyalgia, hypermobile type Ehlers‐Danlos syndrome, hypogammaglobulinemia, intravenous immunoglobulin, joint hypermobility syndrome

## Abstract

Hypermobile type Ehlers‐Danlos syndrome (hEDS) is an underdiagnosed connective tissue disorder characterized by generalized joint hypermobility, chronic fatigue, widespread joints pain, and impaired quality of life. Here, we reported the first hEDS complicated by hypogammaglobulinemia. New insights into classification, diagnosis, and proper management of hEDS are also reviewed.

## 1 INTRODUCTION

In 2017, the International EDS consortium published its new International EDS Classification which now extended EDS to 13 main subtypes.^1^ Hypermobile type Ehlers‐Danlos syndrome (hEDS) is a heritable connective tissue disorder (HCTD) primarily characterized by generalized joint hypermobility (GJH), related musculoskeletal manifestations, and lack of the typical skin/subcutaneous involvement in the classical and vascular types of EDS. hEDS is often associated with other systemic symptoms such as chronic pain, chronic fatigue, dysautonomia, anxiety, depression, sleep disturbance, and overall a poorer quality of life.^2^ As hEDS is the only type of main EDS types that encompass variety of complexities but without no known diagnostic molecular defects which makes it a clinical diagnosis, the diagnostic criteria have been recently refined with the purpose of better identifying the correct patients and differentiating hEDS from other HDCTs with joint hypermobility. Similar to the diagnosis, management of hEDS is also challenging with its complexed presentations that can almost involve any organ system. Here, we are reporting a hEDS patient associated with hypogammaglobulinemia. We also conducted a case‐based literature review of updated classifications of EDS, diagnosis, management, and future directions of hEDS.

## 2 CASE PRESENTATION

A pleasant 54‐year‐old white female was referred to the resident clinic to establish care by her previous primary care physician (PCP). The patient has well‐refined makeup, since the beginning of our encounter, she had to clean the tears mixed with sticky secretion every a few minutes with a napkin, she emphasized she was not crying but has been suffering from severe sinusitis and conjunctivitis in the past 6 months, she has been following up with ENT for recurrent sinusitis and on allergy shot, in addition to this acute distress, she has also been following up with her previous PCP for chronic joints pain associated with fibromyalgia. She also self‐reports her problems are all because of EDS, but she denies previous clinical or genetic diagnosis of any type of EDS. In addition to medical distresses, she recently lost her job which has made her unemployed for the first time in her life, the next day she also lost her health insurance because of which she has been trying to find another PCP in the past a few months. Review of system was positive for watery eyes, multiple joints pain involving ankles, knees, hips, lower back, shoulder, and neck. Negative for fever, chills, cough, short of breath, syncope/near‐syncope episodes, chest discomfort, palpitations, or abdominal discomfort. Her medication list includes vitamin D, vitamin B12, vitamin C, iron tablet, duloxetine 20 mg daily, oxycodone/acetaminophen 10 mg/325 mg q6h prn, tramadol 50 mg bid, and trazodone 50 mg at bedtime. Past medical history includes fibromyalgia and chronic joint pain, denies history of joint subluxations or dislocations. Surgical history is unremarkable. Family history: father and one sister were diagnosed with EDS (unknown type). She currently smokes daily due to stress, not alcoholic, denies any drug abuse.

### 2.1 Vital signs and physical exam

**Figure 1:**
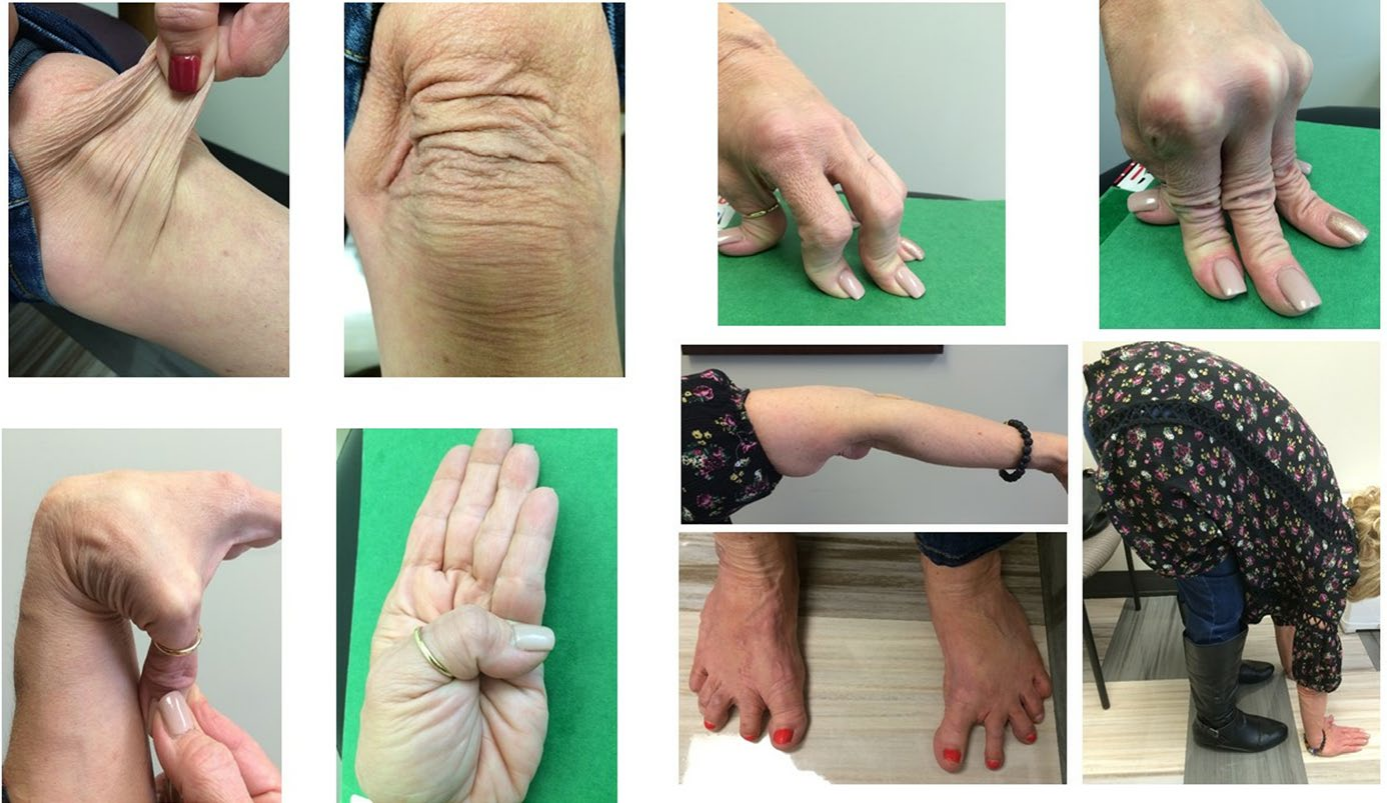
Skin hyperextensibility and Generalized Joint Hypermobility on physical exam

**Figure 2:**
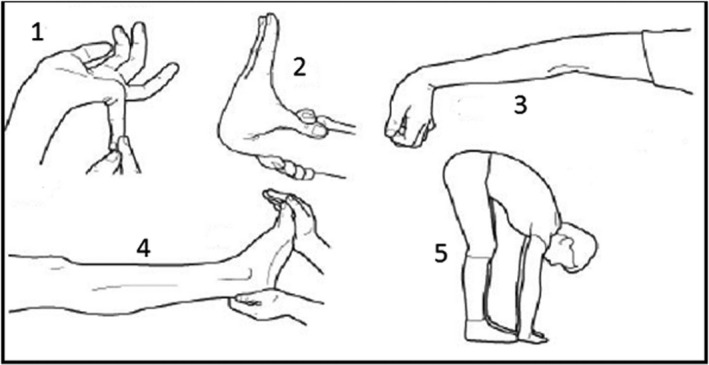
The Beighton Score

Temperature 36.5°C, blood pressure 127/77 mm Hg, pulse 91 bmp, respiratory rate 18 bpm, O2 saturation 98% on room air, weight 52.2 kg, height 155 cm and calculated BMI 21.7. On detailed physical examination, several abnormalities were identified. She has atrophic scar no her nose which is from a dog bite many years ago, mild to moderate midfacial hypoplasia and micrognathia. Skin hyperextensibility is presented on bilateral elbows, forearms, and hands. Sagging and doughy skin folds were presented on bilateral knees. The musculoskeletal examination revealed normal strength in all extremities, with pain to palpation over bilateral ankles/knees/hips, lower back, and back of neck. Joints were examined for hypermobility using the Beighton scale criteria (Figure 2). The patient scored a 5/9: bilateral fifth digit passively extended to 90°, thumb was opposable to the forearm bilaterally, and bilateral elbow extension past 10°. She was able to easily palm the floor without bending her knees. Foot deformities include pes planus, pes vulgus, and hallux varus (Figure 1).

### 2.2 Laboratory

CBC with differential, BMP, thyroid function, and liver function panel are in normal range, HbA1c 5.5%, vitamin B12, and folate levels are in normal range, and vitamin D level was 37 ng/mL (30‐80), lipid panel: cholesterol 213 mg/mL, triglycerides 159 mg/mL, VLDL 32 mg/mL, and LDL 130 mg/dL, rheumatology markers include ANA and ESR are negative. 12‐lead EKG showed normal sinus rhythm, heart rate 92 bpm, normal axis, normal intervals, and no chronic or new ischemic changes.

Clinical diagnosis of Ehlers‐Danlos Hypermobile type was made. A cardiac ECHO was then ordered to rule out valvular and vascular disorders. Gabapentin 100 mg PO tid was added, patient has now established care with pain management clinic, ophthalmology, and physical therapy. We also referred patient back to her previous ENT physician to get retested for possible development of new allergens. She is currently receiving a new course of allergy shots together with eye drops prescribed by ophthalmologist. Her immunoglobulin levels turned out to be low with IgG at 600 mg/dL (ref. 694‐1618). Patient was then referred to hematology and is currently been treated with monthly IVIG supplementation. In the follow‐up appointment, patient’s conjunctivitis and joints pain have been much better controlled, her repeat IgG level was 917 mg/dL, and she feels comfortable to look for new job.

## 3 DISCUSSION

### 3.1 Diagnosis

The Ehlers‐Danlos syndromes are classically defined as a heritable connective tissue disorder (HCTD) characterized by generalized joint hypermobility (GJH), skin hyperextensibility, and tissue fragility affecting skin, ligaments, joints, blood vessels, and inner organs. Over the past decades, EDS has been grouped by six subtypes based on the Villefranche Nosology, with growingly identified novel mutations associated with different types of EDS, in 2017, the International EDS Consortium published the new International EDS Classification with regrouping of 13 subtypes which maintained the previously clinically oriented nomenclature and added a few more types with novel phenotypic patterns.^1^

As a main type of EDS, hEDS is a primarily characterized by generalized joint hypermobility, related musculoskeletal presentations, but lack of the typical skin/subcutaneous involvement in the classical and vascular types of EDS. Though not included into the major diagnostic criteria, hEDS is often associated with systemic symptoms such as chronic pain, chronic fatigue, dysautonomia, anxiety, depression, sleep disturbance and overall a poorer quality of life.^2^ Joint Hypermobility Syndrome (JHS) and hEDS are closely overlapped and often clinically indistinguishable, thus commonly referred at JHS/hEDS.^3^, ^4^ Generalized joint hypermobility (GJH) is typically evaluated by the Beighton score with a Beighton score of ≥5 considered as positive for the presence of GJH (Figure 2).

Hypermobile type Ehlers‐Danlos syndrome currently remains a clinical diagnosis with no “gold standard” laboratory or genetic test to rule in or out the diagnosis. As identifying the pathogenic molecular mutations has become the main route of diagnosis to diagnose other types of EDS, it is expected that genetic test will become available to diagnose hEDS in the near future, for example, studies have identified gene mutation among hEDS families such as TNXB and COL3A1 gene variants. The diagnostic criteria have been recently refined with the purpose of better identifying the correct patients and differentiating hEDS from other HDCTs with joint hypermobility. According to the new classification of the International EDS Consortium, diagnosis of hEDS requires meeting ALL 3 criteria (Table 1, see more details in Ref. 1):

Criterion 1: GJH (a cut‐off Beighton score ≥6 for prepubertal children and adolescents, ≥5 for pubertal men and women up to the age of 50, and ≥4 for those >50 years of age), minor adaptations and assessment of alternative joints are also suggested in certain situations (eg, age, acquired joint imitations due to injury/surgery etc).
Criterion 2: Two of more of the following features: (a) systemic manifestations of generalized connective tissue disorder, (b) positive family history, (c) Musculoskeletal complications (chronic pain >3 months and/or recurrent joint dislocations/instability).
Criterion 3: absence of unusual skin fragility as of other types of EDS and exclusion of alternative heritable/acquired connective tissue disorder including autoimmune rheumatologic conditions.

**Table 1:** Diagnosis criteria of joint hypermobility syndrome and hypermobile type Ehlers‐Danlos syndrome (hEDS)^22^

Hypermobility syndrome	Ehlers‐Danlos (hypermobility type) (hEDS)
**Brighton criteria**	**Villefranche** **criteria**
Major criteria	Major criteria
Generalized joint hypermobility (Beighton score ≥4: currently or historically)	Generalized joint hypermobility (Beighton score ≥5)
Arthralgia (≥4 joints, ≥3 mo)	Skin involvement (hyperextensibility: smooth, soft and velvety skin)
Minor criteria	Minor criteria
Beighton score of 1, 2, and 3 (0,1,2, or 3 if aged ≥50 y)	Recurring joint dislocations
Arthralgia in 1, 2, or 3 joints or back pain, spondylosis, spondylolysis, or spondylolisthesis	Chronic joint and limb pain (≥3 mo)
Dislocation/subluxation in more than one joint or in one joint on more than one occasion	Positive family history
Soft tissue rheumatism: >3 lesions (eg, epicondylitis, tenosynovitis, bursitis)	
Marfanoid habitus: tall, slim, span/height ratio >1.03, upper: lower segment ratio <0.89, arachnodactyly	
Abnormal skin: striae, hyperextensibility, thin skin, papyraceous scarring	
Eye signs: Drooping eyelid or myopia or antimongoloid slant	
Varicose veins, hernia, or uterine/rectal prolapse	

Diagnosis of hEDS in our patient was made after carefully reviewing the above criteria, the patient has fully met criterion 1 and 3, she fairly met criterion 2 with self‐reported positive family history of father and a sister diagnosed with EDS for which we were unable to obtain detailed documentation (2a) and chronic joint pain (2b) as well as several features that fall into criterion 2c. Meanwhile, we have considered alternative diagnosis, but have ruled out classical EDS or classical‐like EDS (no classical skin fragility and bruising), cardiac/vascular EDS (no history of arterial/colon rupture/dissection or spontaneous pneumothorax) or arthrochalasia EDS (no history of congenital hip dislocation).

### 3.2 Management

Management of hEDS includes relief of both acute and chronic symptoms and primary and secondary prevention of complications. Cardiac assessment including echocardiography needs to be considered to look for aortic root dilation and mitral valve prolapse. Surgical complications in hEDS are generally less than other types of EDS particularly the classical and vascular type, but being more gentle during operations, careful sutures and longer suture in‐place (twice as long as normal population) are generally recommended to ensure wound heal and tissue recovery.^5^ Ascorbic acid (2 g/d for adults) may reduce bruising. Deamino‐Delta‐D‐Arginine Vasopressin (DDAVP) may be useful to normalize bleeding time. Free downloadable guidelines for peri‐surgical management for EDS is available at the OrphanAnesthesia website (http://www.orphananesthesia.eu/en/rare-diseases/published-guidelines/cat_view/61-rare-diseases/60-published-guidelines/89-ehlers-danlos-syndrome.html).

Joint pain is one of the most common chronic symptoms which can give acute exacerbations and is often combined with chronic fatigue. hEDS may often be misdiagnosed as fibromyalgia because of diffuse pain, but they are considered as two distinct conditions. Studies reported that pain related to hEDS shares similar mechanisms underlying fibromyalgia but the physical and functional impact of EDS pain is worse than that of rheumatoid arthritis.^6^

Multiple specialists are often needed including pain clinic, psychiatry, orthopedics, physical therapy, yoga/massage, and exercise program, etc.^7^ Arthur et al conducted a survey on the pain control methods used among 1179 EDS patients, and participants reported the most helpful methods for acute pain control included opioids, surgical interventions, splints and braces, heat therapy, nerve blocks, and physical therapy, while chronic pain was most effectively controlled with opioids, heat therapy, splints or braces, and surgical interventions. The overall goal should be controlling pain to a tolerable range rather than completely pain‐free.^8^

Scheduled regimen of multiple medications is often more effective than as‐needed use of one or two medications. Nonopioid oral analgesics such as acetaminophen, nonsteroidal anti‐inflammatory (NSAID) or cyclooxygenase‐2 (COX2) inhibitor should be maximized first. Topical agents and muscle relaxants can also be helpful.^7^, ^9^ Benzodiazepines should be used only cautiously for short‐term and are poor choices for long‐term use, particularly risky when coprescribed with opioids.^10^ For patients with neuropathic pain, regiment can include a tricyclic antidepressant, serotonin‐norepinephrine reuptake inhibitor, and/or an anti‐epileptic agent. Opioids are rarely needed for the treatment of chronic musculoskeletal pain^7^ and are not recommended for neuropathic pain.^11^ Narcotics (Opioids and tramadol) should be reserved for acute pain episodes or for those who are inadequately managed after trial of all of the above approaches and should be added on to the above regimen in the lowest effective doses rather than replacing nonopioids. Complications should be discussed with patients and closely monitored which include increased risk of GI ulcers/bleeding with NSAIDS/COX inhibitor, increased risk of coronary artery disease and renal insufficiency with chronic high dose NSAIDS, and signs of serotonin syndrome with medications that increase serotonin levels etc.^2^

In the United States, IVIG has been indicated or under investigations for relieving pain associated with chronic inflammatory demyelinating polyneuropathy (CIDP), Stiff‐Person syndrome, postpolio syndromes, pain in sickle cell disease and refractory neuropathic pain syndrome.^12^, ^13^, ^14^, ^15^, ^16^ A significant subgroup of fibromyalgia patients has findings indicative of chronic inflammatory demyelinating polyneuropathy (CIDP) and these patients had significant improvement with IVIG treatment.^17^ Hypogammaglobulinemia in patient with fibromyalgia was previously reported,^18^ however, it has not been reported in patients with EDS. The current case report particularly raised the interest finding of hypogammaglobulinemia in combination with hEDS and fibromyalgia, and either this represents an accidental finding or there is underlying pathophysiological association will need future studies to confirm. Our patient also suffered from recurrent sinusitis and conjunctivitis for more than 6 months, with the hope to relieve the symptoms and prevent frequent recurrence, our patient was referred to hematologist and started on monthly IVIG therapy.

With the allergic symptoms (rhinitis and conjunctivitis), mast cell activation syndrome is in the differential diagnosis,^19^ the patient has had no other systemic signs and symptoms such as skin flushing/pruritus/angioedema, anaphylaxis or gastrointestinal symptoms (diarrhea/cramping/bloating). The patient needs to be followed up and monitored for these symptoms. If indicated, laboratory assessments include serum tryptase/heparin/chromogranin A level, 24‐hours urinary histamine metabolite (N‐methylhistamine)/prostaglandin and tissue biopsy to look for CD25 and CD2 co‐expression on CD 117+ Cells. However, MCAD is currently incurable and treatment is symptomatic. Environmental, medical and emotional triggers should be avoided, higher dose of longer acting, nonsedating second‐generation H1 antihistamine (eg, cetirizine, fexofenadine, and loratadine) bid or tid are considered more beneficial, and chronic glucocorticoid therapy is not recommended. Adding MC‐stabilizing agents such as sodium cromoglicate and ketotifen to antihistamines is also alternative approach. From the few reports available, nonsteroidal immunosuppressants such as cyclophosphamide, cyclosporine, azathioprine, and monoclonal antibodies such as omalizumab^20^ and alemtuzumab are only occasionally helpful.^21^

## 4 CONCLUSION AND FUTURE DIRECTIONS

Though hEDS remains the only type of main EDS types that can present with variety of complexities but without known diagnostic molecular defects. Multiple genetic factors have been reported to be associated with different phenotypes. In addition to presentations that are currently included in the diagnostic criteria, emerging interesting and/or controversial features that have been reported but need more large‐scale investigations include dysautonomia, poor craniocervical junction stability, mast cell activation disorders (MCAD), gastrointestinal symptoms and abnormal bone density etc. Here, we reported a hEDS patient with chronic joint pain, hypogammaglobulinemia and recurrent conjunctival‐sinusitis. Patient’s quality of life has been significantly improved with a multispecialty management. Future research directions include more complete collection of phenotypic features, subgrouping of hEDS patients with unique phenotypic patterns, and define their genetic etiologies. Researches and trials are also greatly needed to better optimizing the management of patients’ acute and chronic complications.

## CONFLICT OF INTEREST

None declared.

## AUTHOR CONTRIBUTION

WZ and DOT: conducted the clinical examination, literature research, analysis of clinical records, and wrote the manuscript. RJ and KW: reviewed the clinical records and provided expert consultations. WZ, KW, RJ and DOT: approved the final version of the manuscript.
